# Efficacy of T-705 (Favipiravir) in the Treatment of Infections with Lethal Severe Fever with Thrombocytopenia Syndrome Virus

**DOI:** 10.1128/mSphere.00061-15

**Published:** 2016-01-06

**Authors:** Hideki Tani, Aiko Fukuma, Shuetsu Fukushi, Satoshi Taniguchi, Tomoki Yoshikawa, Naoko Iwata-Yoshikawa, Yuko Sato, Tadaki Suzuki, Noriyo Nagata, Hideki Hasegawa, Yasuhiro Kawai, Akihiko Uda, Shigeru Morikawa, Masayuki Shimojima, Haruo Watanabe, Masayuki Saijo

**Affiliations:** a Department of Virology I, National Institute of Infectious Diseases, Tokyo, Japan; b Department of Pathology, National Institute of Infectious Diseases, Tokyo, Japan; c Division of Experimental Animal Research, National Institute of Infectious Diseases, Tokyo, Japan; d Department of Veterinary Science, National Institute of Infectious Diseases, Tokyo, Japan; e National Institute of Infectious Diseases, Tokyo, Japan; Boston University

**Keywords:** SFTS, favipiravir, efficacy, infection, type I interferon, T-705

## Abstract

Severe fever with thrombocytopenia syndrome virus (SFTSV) is the causative agent of SFTS, an emerging hemorrhagic fever. This disease has a high case fatality rate and is endemic to China, South Korea, and Japan. Because there are currently no effective therapeutics for SFTS, potent and safe antivirals are needed for the treatment of SFTS. The inhibitory effect of T-705 (favipiravir) on the replication of SFTSV in Vero cells was evaluated. Mice lacking the type I interferon receptor (IFNAR^−/−^) were used as an *in vivo* lethal model for SFTSV infection. T-705, which has been licensed as an anti-influenza drug in Japan, inhibits SFTSV replication both *in vitro* and *in vivo*. T-705 inhibited replication of SFTSV in Vero cells by 5 log units, with a 50% inhibitory concentration (IC_50_) and IC_90_ of 6.0 µM and 22 µM, respectively. Intraperitoneal or oral administration of T-705 for 5 days to IFNAR^−/−^ mice infected with lethal SFTSV significantly improved survival rates (100% survival) without causing body weight loss and reduced the viral load in the serum. Ribavirin also inhibited SFTSV replication. However, it was less effective than T-705 both *in vitro* and *in vivo*. A time-of-drug-addition study revealed that therapeutic T-705 treatment of SFTSV infection in IFNAR^−/−^ mice was effective. These results suggest that T-705 is a promising candidate for the treatment of SFTS.

**IMPORTANCE** Severe fever with thrombocytopenia syndrome (SFTS), caused by SFTS virus (SFTSV), is a recently identified emerging viral infectious disease. Despite the medical importance of this disease, there are currently neither vaccines nor effective therapeutics for SFTS. T-705, which is a pyrazine derivative, has shown broad antiviral activity against various RNA viruses. The present study demonstrated, for the first time to our knowledge, the efficacy of T-705 in treating SFTSV infection in a mouse lethal model. T-705 showed a high efficacy in the treatment of SFTSV infection in the mouse model, even when treatments were initiated after onset of the disease.

## Abstract (precis)

Severe fever with thrombocytopenia syndrome (SFTS), caused by SFTS virus (SFTSV), is a recently identified emerging viral infectious disease. Despite the medical importance of this disease, there are currently neither vaccines nor effective therapeutics for SFTS. T-705, which is a pyrazine derivative, has shown broad antiviral activity against various RNA viruses. The present study demonstrated, for the first time to our knowledge, the efficacy of T-705 in treating SFTSV infection in a mouse lethal model. T-705 showed a high efficacy in the treatment of SFTSV infection in the mouse model, even when treatments were initiated after onset of the disease.

## INTRODUCTION

Severe fever with thrombocytopenia syndrome (SFTS) is a recently discovered emerging infectious disease which is epidemic in China (1), South Korea (2), and Japan (3). The causative agent of SFTS is a novel phlebovirus of the family *Bunyaviridae*; it has been designated SFTS virus (SFTSV). The major clinical manifestations of SFTS are rapid onset of high fever, gastrointestinal tract symptoms, hemorrhagic tendency, and thrombocytopenia and leukopenia in the total blood cell counts. SFTS occurs throughout the year, but most patients become ill between spring and autumn; the case fatality rate can be as high as 30% in Japan. Although effective treatments are required, there are currently no safe and effective antivirals or other therapies for SFTS.

Ribavirin is an effective antiviral drug against several RNA virus infections in animal models, including bunyaviruses, such as Andes virus (4, 5) and Rift Valley fever virus (6). Ribavirin has also been shown to have an inhibitory effect on SFTSV replication *in vitro* (7) and a partial effect *in vivo* (8). However, it has not been possible to demonstrate a beneficial effect of ribavirin in the treatment of hospitalized patients with SFTS in China (9, 10).

The establishment of animal models is necessary for the evaluation of both antivirals and vaccines for SFTSV infection. Adult mice and hamsters are not susceptible to SFTSV infection (11). In nonhuman primate models, rhesus macaques showed mild symptoms similar to those of SFTS in humans (12). Mice lacking the type I interferon receptor (IFNAR^−/−^) on the 129X1/Sv background have been shown to be a useful *in vivo* lethal animal model for SFTSV infection (8, 13).

T-705 (favipiravir [Avigan]; 6-fluoro-3-hydroxy-2-pyrazinecarboxamide) was developed by Toyama Chemical Co., Ltd., and is a pyrazine derivative. It has a broad spectrum of activity against various RNA viruses, such as the *Orthomyxoviridae* (seasonal influenza viruses as well as highly pathogenic and oseltamivir-resistant strains) (14–16), *Picornaviridae* (poliovirus and rhinovirus) (17), *Flaviviridae* (West Nile virus and yellow fever virus) (18, 19), *Togaviridae* (Western equine encephalitis virus and Chikungunya virus) (20, 21), *Arenaviridae* (Lassa virus, Junin virus, Pichinde virus, Guanarito virus, and Machupo virus) (22–26), and *Filoviridae* (Ebola virus) (27, 28). T-705 is also highly effective against members of the family *Bunyaviridae*, including La Crosse, Rift Valley fever, sandfly fever, Andes, and Crimean-Congo hemorrhagic fever (CCHF) viruses (22, 23, 29, 30). The antiviral activity of T-705 is stronger than that of ribavirin in *in vitro* and *in vivo* studies (31). The related pyrazinecarboxamides T-1105 and T-1106, which were also discovered and synthesized by Toyama Chemical Co., Ltd., have also been shown to be effective against several pathogenic RNA virus infections (23, 31).

In the present study, we investigated the inhibitory effects of T-705 and the related pyrazinecarboxamides T-1105 and T-1106 in a cell culture model for SFTSV infection. Furthermore, we examined the efficacy of T-705 in the treatment of SFTSV infections using a lethal mouse model for SFTS. The efficacy of T-705 in *in vitro* and *in vivo* studies was compared with that of ribavirin.

## RESULTS

### *In vitro* antiviral activity of T-705 against SFTSV.

**FIG 1:**
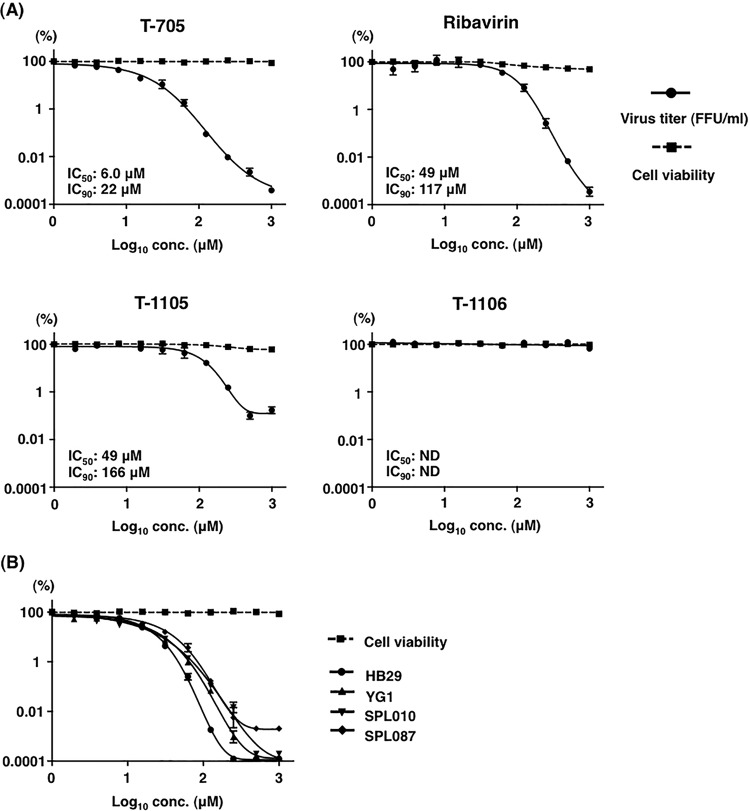
Inhibitory effect of T-705, ribavirin, T-1105, and T-1106 on SFTSV replication in Vero cells. (A) Vero cells were pretreated with various concentrations of T-705, ribavirin, T-1105, and T-1106 4 h before infection and were inoculated with SFTSV (SPL010) at an MOI of 0.1. (B) Vero cells were pretreated with various concentrations of T-705 and infected with HB29, YG1, SPL010, or SPL087 strains of SFTSV at an MOI of 0.1. The virus infectious dose in the culture supernatants and cell growth and viability after treatment with each compound are shown. A sigmoidal dose-response curve was fitted to the data using GraphPad Prism6 (GraphPad Software).

The antiviral activity of T-705 against the SFTSV strain SPL010 was evaluated in Vero cells in parallel with ribavirin, T-1105, or T-1106. T-705, ribavirin, and T-1105 inhibited replication of SFTSV by approximately 5 or 3 log units at a concentration of 1,000 µM (Fig. 1A), whereas T-1106 showed no inhibitory effect on viral replication at the same concentration. The 50% and 90% inhibitory concentrations (IC_50_ and IC_90_, respectively) of T-705 were 6.0 µM and 22 µM, respectively. T-705 as well as T-1105 and T-1106 did not affect cell viability in the test range, as measured by a WST cell viability assay (Fig. 1). The antiviral activity of T-705 against various strains of SFTSV was also evaluated (Fig. 1B). T-705 inhibited not only the replication of Japanese strains, including YG1 and SPL087, but also the replication of the Chinese strain HB29.

### *In vivo* efficacy of T-705 against SFTSV infection in IFNAR^−/−^ mice.

**FIG 2:**
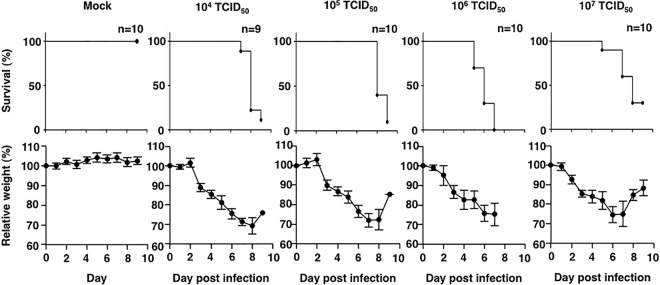
Survival and sequential body weight of IFNAR^−/−^ mice infected with different doses of SFTSV. Nine or ten female mice in each group were inoculated s.c. with 1.0 × 10^4^, 1.0 × 10^5^, 1.0 × 10^6^, or 1.0 × 10^7^ TCID_50_ of SFTSV (SPL010). Ten female mice not infected with SFTSV acted as the control group (mock). Survival was determined using Kaplan-Meier analysis and GraphPad Prism6. Relative weight was calculated and is shown as means with standard deviations.

Before the efficacy of the treatment against SFTSV infections was tested in the IFNAR^−/−^ C57BL/6 mouse model, the optimal lethal infection dose for SFTSV strain SPL010 was determined. Furthermore, disease symptoms, including loss of body weight, were characterized. The IFNAR^−/−^ mice were subcutaneously (s.c.) infected with 1.0 × 10^4^, 1.0 × 10^5^, 1.0 × 10^6^, or 1.0 × 10^7^ 50% tissue culture infective doses (TCID_50_) of SFTSV. Infection with 1.0 × 10^4^ and 1.0 × 10^5^ TCID_50_ resulted in death of most of the IFNAR^−/−^ mice 7 to 8 days postinfection. All the mice infected with 1.0 × 10^6^ TCID_50_ of SFTSV died after 5 to 7 days postinfection (Fig. 2). Conversely, the survival rate was higher in the mice infected with 1.0 × 10^7^ TCID_50_ than in those infected with the lower dose. Before death, most mice in all of the groups lost weight. Indeed, some of them lost more than 20% of their weight, but some recovered (Fig. 2). Consequently, in the experiments described below, the acceptable endpoint limit was set to 30% weight loss. A lethal outcome was consistently observed in the mice infected with 1.0 × 10^6^ TCID_50_ of SFTSV.

**FIG 3:**
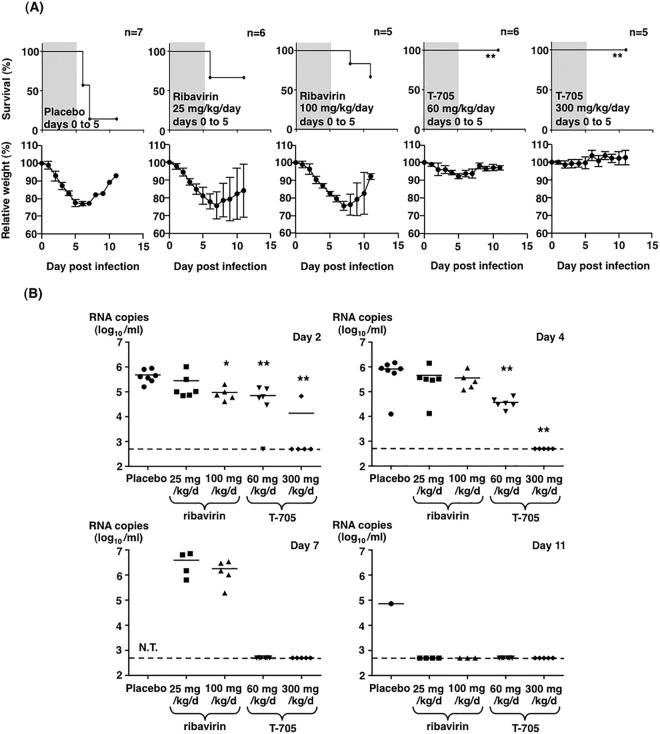
Treatment of SFTSV-infected IFNAR^−/−^ mice with T-705 or ribavirin. (A) Five to seven male mice in each group were inoculated s.c. with 1.0 × 10^6^ TCID_50_ of SFTSV (SPL010). Mice were treated with ribavirin at a dose of 25 or 100 mg/kg/day or T-705 at a dose of 60 or 300 mg/kg/day. The drugs were administered intraperitoneally once daily. Placebo mice received the same volume of PBS. Treatment was continued for 5 days as indicated in the upper columns (shaded in gray with the survival curves). Survival was determined using Kaplan-Meier analysis and GraphPad Prism6. Relative weight is shown as means with standard deviations. (B) SFTSV RNA levels in blood samples collected at 2, 4, 7, or 11 days postinfection were determined by quantitative RT-PCR assays. One-way ANOVA with Bonferroni’s multiple-comparison test was used to determine the level of statistical significance. Dashed lines indicate the detection limits of the assay in blood samples. Significance was determined in comparison to the results of the placebo group: **, *P* < 0.01; *, *P* < 0.05; N.T., not tested.

**FIG 4:**
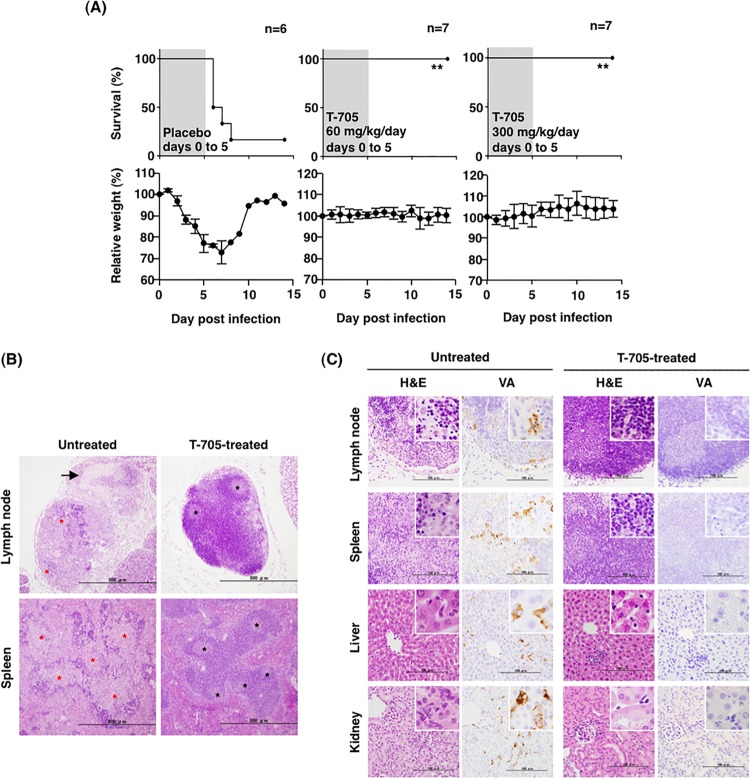
T-705 treatment of SFTSV-infected IFNAR^−/−^ mice through oral administration. (A) Six or seven female mice in each group were inoculated s.c. with 1.0 × 10^6^ TCID_50_ of SFTSV (SPL010). Mice were treated with T-705 at a dose of 60 or 300 mg/kg/day. The drug was administered once daily p.o. using a stomach probe. Placebo mice received 2.9% sodium bicarbonate solution as a solvent for T-705. T-705 was administered for 5 days as indicated in the upper columns (shaded in gray with the survival curves). Survival was determined using Kaplan-Meier analysis and GraphPad Prism6. **, *P* < 0.01 compared with the results of the placebo group. Relative weight is shown as means with standard deviations. (B and C) Histopathological and immunohistochemical examinations of the tissues collected from the SFTSV-infected IFNAR^−/−^ mice treated with T-705 or left untreated. Tissues were collected from mice at 4 days postinfection (untreated mice) or at 12 days postinfection (T-705-treated mice). (B) Low-power views of the cervical lymph node and the spleen. Bars, 500 µm. The arrow (upper left) indicates necrosis; blask asterisks indicate intact structures; red asterisks indicate necrosis. (C) Histopathology and immunohistochemical analyses of cervical lymph nodes, spleens, livers, and kidneys. Bars, 100 µm. H&E, hematoxylin-and-eosin staining; VA, viral antigens detected by immunohistochemistry.

Next, the efficacy of T-705 in the postexposure treatment was examined using the IFNAR^−/−^ mice infected with SFTSV in comparison with those of the ribavirin and placebo groups (Fig. 3A). The doses and experimental conditions of ribavirin and T-705 administration were determined as was done in previous studies (16, 31). The efficacies of these drugs were evaluated on the basis of the survival rate at 11 days postinfection. Intraperitoneal (i.p.) administration of T-705 at a dose of 60 or 300 mg/kg/day for 5 days commenced 1 h postinfection, and this completely protected the mice from death upon SFTSV infection. Although the body weights of the mice treated with 60 mg/kg/day of T-705 decreased slightly until 5 days postinfection, those of the 300 mg/kg/day-treated mice did not. The survival rate of mice treated with ribavirin significantly increased compared with that of the placebo-treated mice. However, approximately 40% of the mice treated with ribavirin at a dose of 25 or 100 mg/kg/day lost body weight more notably than those treated with T-705. Ribavirin-treated mice started to die at 6 days postinfection. Oral administration of T-705 at a dose of 60 or 300 mg/kg/day also completely protected mice from lethal infection (Fig. 4A).

To examine the viral load in mice treated with these drugs, SFTSV RNA levels in the blood were determined by a quantitative RT-PCR (qRT-PCR) assay (32). The RNA levels slightly increased until 4 or 7 days postinfection in the mice treated with 25 mg/kg/day ribavirin, 100 mg/kg/day ribavirin, and placebo. In contrast, in the blood of the mice treated with 300 mg/kg/day T-705, viral RNA was almost undetectable at 2 days postinfection. Furthermore, the viral RNA was undetectable in the blood of the mice treated with 60 mg/kg/day T-705 at 7 days postinfection (Fig. 3B).

### Histopathology of IFNAR^−/−^ mice infected with SFTSV.

Histopathological and immunohistochemical analyses were performed with the placebo- and T-705-treated mice. The cervical lymph nodes of the T-705-treated mice exhibited an intact lymphoid architecture (Fig. 4B, upper right). In contrast, massive necrosis was induced in the cervical lymph nodes of the placebo-treated mice (Fig. 4B, upper left). Furthermore, corticomedullary demarcation was unclear, and lymphocytes were significantly deleted (Fig. 4B, upper left).

The spleens of the T-705-treated mice showed intact histopathology (Fig. 4B, lower right), although the spleens of the placebo-treated mice were depleted of lymphocytes in the white pulps, and the architecture of the follicles was not conserved (Fig. 4B, lower left).

In addition to the cervical lymph nodes and spleens, no obvious pathological changes were observed in the liver and kidneys of the T-705-treated mice (Fig. 4C, right). There were no viral-antigen-positive cells in any of the examined organs of the T-705-treated mice (Fig. 4C, right, right column). In contrast, depletion of lymphocytes with apoptosis was demonstrated in the cervical lymph nodes and the white pulp of the spleens of the placebo-treated mice (Fig. 4C, left, upper and second left panels). Immunohistochemical analysis revealed that some viral-antigen-positive cells were present in these lesions of the placebo-treated mice (Fig. 4C, left, upper and second right panels). Diffuse infiltrations of inflammatory cells, such as neutrophils and swollen Kupffer cells, were markedly elevated in the liver sinuses of the placebo-treated mice (Fig. 4C, left, third left panel). Morphologically identified Kupffer cells in the placebo-treated mice were also positive for the viral antigen (Fig. 4C, left, third right panel). Large mononuclear cells, which were also positive for viral antigen, resided in the microvessels in the kidney of the placebo-treated mice (Fig. 4C, left, lower left, and right panels). Focal necrosis with slight inflammatory cell infiltration was observed in the livers of the placebo-treated mice (Fig. 4C, right, third left panel).

### T-705 therapeutic study.

**FIG 5:**
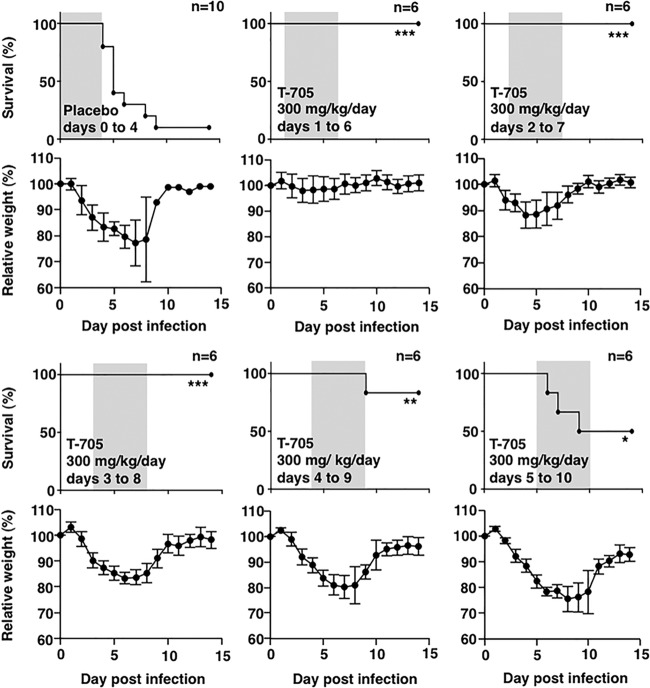
Influence of the time from challenge to the initiation of the T-705 treatment on SFTSV infections in the IFNAR^−/−^ mice. Six or 10 male mice in each group were inoculated s.c. with 1.0 × 10^6^ TCID_50_ of SFTSV (SPL010). Mice were treated with T-705 at a dose of 300 mg/kg/day. Treatment was commenced 1, 2, 3, 4, or 5 days postinfection. T-705 was administered once daily until death or for 5 days. Survival was determined using Kaplan-Meier analysis and GraphPad Prism6. Significance was determined relative to results for the placebo group: ***, *P* < 0.001; **, *P* < 0.01; *, *P* < 0.05. Relative weight is shown as means with standard deviations.

To determine the therapeutic efficacy of T-705 in the treatment of SFTSV infection, the IFNAR^−/−^ mice were intraperitoneally administered T-705 at a dose of 300 mg/kg/day starting at various days after SFTSV challenge (Fig. 5). All T-705-treated mice survived lethal SFTSV infection when the treatment was initiated on or earlier than 3 days postinfection. The mice treated with T-705 from 4 and 5 days postinfection exhibited over 50% survival, although the mice were very ill, with over 15% weight loss.

## DISCUSSION

T-705 inhibited SFTSV replication *in vitro* and showed therapeutic efficacy *in vivo*. T-705 has been shown to possess favorable antiviral activities against various RNA viruses and has a therapeutic effect on these viral infections in animal models (16, 31). T-705 was identified as a potential drug for the treatment of influenza virus infection. Regarding the mechanism of action of T-705, it has been reported that T-705 is converted to its phosphoribosylated metabolites (T-705RMP and T-705RTP) by host cellular kinases and that T-705RTP inhibits the activity of the RNA-dependent RNA polymerase of a number of viruses (33). Recently, T-705 has been licensed for use by the Ministry of Health, Labour and Welfare of Japan. However, its use has been limited to the events of the outbreak due to novel or reemerging influenza viral infections. T-705 was also considered as a potential therapeutic agent against Ebola virus disease (EVD) during the 2014-2015 EVD outbreak throughout West Africa (34).

Therapeutic options for SFTS tested so far include administrations of ribavirin, steroids, and/or plasma exchange in severely ill patients (9, 35). However, there are no specific therapeutics which have been proven to be effective for SFTS. Serum possessing neutralizing activity against SFTSV has been collected from convalescent-phase SFTS patients and was shown to have treatment capability using a mouse model of SFTSV infection (8). Administration of ribavirin also showed therapeutic efficacy in the mouse model, but the efficacy was less than that of the convalescent-phase SFTS patient serum (8). Administration of ribavirin was confirmed to be partially protective against lethal infection of the IFNAR^−/−^ mice with SFTSV (Fig. 1).

In an *in vitro* assay, the inhibitory effect of T-705 on SFTSV replication was higher than that of ribavirin, T-1105, and T-1106 in the cells used. It is difficult to compare the effect of T-705 on various RNA viruses, because the IC_50_ and 50% cytotoxic concentration, at which cell viability was reduced by 50% (CC_50_), of T-705 depend on the cells used for the assay or the experimental methods required to determine viral titers. Notwithstanding this limitation, the IC_50_ of T-705 for SFTSV (IC_50_: 6.0 µM, less than 1.0 µg/ml) is lower than those reported for other RNA viruses, including *Arenaviridae*, *Bunyaviridae* (excluding SFTSV), *Filoviridae*, *Flaviviridae*, *Picornaviridae*, and *Caliciviridae* (16). Therefore, it is suspected that the inhibitory effects of T-705 on SFTSV are stronger than those on other viruses.

Several background strains of IFNAR^−/−^ mice, including C57BL/6, are highly susceptible to infection with various viruses, including SFTSV (13, 26, 27, 30). The C57BL/6-based IFNAR^−/−^ mice are also susceptible to SFTSV infection, but it was not completely lethal in the present study. The exact mechanism underlying the less-than-100% lethality was not elucidated. However, it was considered to be due to the following mechanisms: (i) the virulence of SFTSV for the IFNAR^−/−^ mice was milder than that of other viruses; (ii) other survival mechanisms except for the interferon-inducing pathway were induced in the IFNAR^−/−^ mice by the infection with SFTSV. Although the relative weight loss in the mice infected with 1.0 × 10^7^ TCID_50_ SFTSV was similar to that of the mice infected with 1.0 × 10^6^ TCID_50_ SFTSV or a lower dose, the survival rate of the mice infected with 1.0 × 10^7^ TCID_50_ SFTSV was higher. Furthermore, the time from virus challenge to death in mice was longer than those in mice infected with lower doses of SFTSV. The viruses used in this study were diluted from a stock virus at 1.0 × 10^8^ TCID_50_/ml and used in both *in vitro* and *in vivo* studies as a same-stock virus. The evidence should be confirmed by further experiments using the same or higher concentrations of SFTSV. However, this phenomenon is not thought to be due to the conditions of mice or technical problems. Innate immune responses through pathways other than the interferon-signaling pathway may be induced by larger amounts of antigens derived from virions in the mice infected with 1.0 × 10^7^ TCID_50_ SFTSV. A similar phenomenon was observed for Ebola virus infection in mice (36).

During pathological examination of the SFTSV-infected IFNAR^−/−^ mice, severe necrotizing lymphadenitis with concurrent depletion of lymphocytes in the cervical lymph node was demonstrated. This was similar to the lesion observed in SFTS patients (3). The histopathological features observed in the lymph nodes, spleens, livers, and kidneys of the SFTSV-infected IFNAR^−/−^ mice were similar to those observed in the same-background immunocompetent C57BL/6 mice (11). In contrast, no pathological lesions were detected in the tissues of CD-1 IFNAR^−/−^ mice infected with SFTSV (13). This discrepancy may be attributable to the difference in the mouse strain, virus strain, and/or experimental approach. More appropriate experimental animals, including genetically modified mice which show the pathological effects of SFTS in humans, should be considered for the evaluation of the pathophysiology and efficacies of developed drugs and vaccines against SFTSV infections.

Comparing the postinfection efficacies of T-705 to that of ribavirin against SFTSV infection in the mouse model, T-705 was more effective than ribavirin in terms of the survival rate and disease progression, including weight loss (Fig. 3A). The viral RNA level in the blood of the T-705-treated mice was lower than that observed in the ribavirin-treated mice at 2 days postinfection (Fig. 3B). No abnormal histopathology was detected in the lymph nodes, spleens, livers, and kidneys of the SFTSV-infected mice treated immediately with T-705 (Fig. 4B). These results indicate that T-705 is more effective in the treatment of the SFTSV-infected mice than ribavirin.

T-705 is an orally administered drug approved for clinical use in Japan. Although T-705 was mainly administered through the i.p. route in the present study, oral administration of T-705 showed a similar efficacy to that of i.p. administration in the mouse model (Fig. 4). The blood concentration of T-705 through i.p. administration was reported to be similar to that observed for oral administration (37).

The administration of T-705 after the onset of the disease did not show efficacy in the treatment of Ebola virus or CCHF virus infections in animal models (27, 30). In contrast, the administration of T-705 in mice 4 to 5 days after SFTSV challenge did show efficacy (Fig. 5). T-705 was effective not only for prophylactic use but also for the treatment of SFTSV infections in the mouse model. The present study confirmed the concept of an antiviral drug being administered as early as possible, indicating that rapid diagnosis of SFTS is required for the rapid therapeutic intervention for this disease.

In summary, T-705 possessed an inhibitory effect on SFTSV replication *in vitro* and also significant efficacy in the treatment of SFTSV infections *in vivo.* To our knowledge, these observations are being reported for the first time. T-705 is a promising candidate drug for the treatment of SFTS in humans.

## MATERIALS AND METHODS

### Ethics statement.

All experiments associated with animals were performed in animal biological safety level 3 (BSL-3) containment laboratories at the National Institute of Infectious Diseases in Japan (NIID) under strict regulations of the animal experimentation guidelines of the NIID. The protocol was approved by the Institutional Animal Care and Use Committee of the NIID (no. 114125 and 215024).

### Cells and viruses.

Vero cells obtained from American Type Culture Collection (Summit Pharmaceuticals International, Japan) were used and maintained in Dulbecco’s modified Eagle’s medium (DMEM) supplemented with 10% heat-inactivated fetal bovine serum (FBS) and antibiotics. The Chinese SFTSV strain HB29 was provided by Mifang Liang and Dexin Li (National Institute for Viral Disease Control and Prevention, China) while the Japanese SFTSV strains YG-1, SPL010, and SPL087 were also used (3). The Japanese strain SPL010, which was mainly used in this study, was isolated from a 62-year-old man who died in 2005. All viruses were propagated in Vero cells, and their infectious doses were determined by the method of limiting dilution assay as previously described (7). The virus stocks were stored at −80°C until use in *in vitro* and *in vivo* experiments. All work with infectious SFTSV was performed in BSL-3 containment laboratories in the NIID, in accordance with the institutional biosafety operating procedures.

### Antiviral compounds.

T-705, T-1105, and T-1106 (Toyama Chemical Co., Ltd., Toyama, Japan) and ribavirin provided by Yamasa Corporation (Chiba, Japan) were used. T-705 and T-1106, T-1105, and ribavirin were dissolved in 2.9% sodium bicarbonate solution, dimethyl sulfoxide (DMSO), and phosphate-buffered saline (PBS), respectively, and used for both *in vitro* and *in vivo* experiments.

### Virus yield reduction assays.

Vero cells were infected with each of the Japanese and Chinese SFTSV strains at a multiplicity of infection (MOI) of 0.1 per cell in the presence of serially 2-fold diluted T-705, T-1105, T-1106, or ribavirin and cultured for 3 days. Supernatants were then collected from the cell cultures. The virus infectious dose of each supernatant was determined by a focus-forming assay. Briefly, Vero cell monolayers were infected with each 10-fold serially diluted viral supernatant and cultured for 2 days with DMEM containing 10% FBS and 1% methylcellulose. After fixation with 10% formalin for 1 h, cells were incubated with rabbit polyclonal antibody to SFTSV NP (#75) (3) for 1 h. Then, the cells were treated with goat anti-rabbit Alexa Fluor 488 (Invitrogen). Focus-forming units (FFU) were determined by counting visible foci. IC_50_ and IC_90_ were calculated using regression analysis.

Cytotoxicity of the drugs tested were measured as described previously (7). Vero cells were cultured for 3 days in the presence of the drugs at designated concentrations without infection with the virus. Cell viability was measured using the cell proliferation reagent WST-1 (Roche Life Science, Penzberg, Germany) according to the manufacturer’s protocol. Cell viability was calculated as follows: [(absorbance of cells in the presence of the drug − absorbance of no cells in the absence of the drug)/(absorbance of cells in the absence of the drug − absorbance of no cells in the absence of the drug)] × 100.

### Animals.

IFNAR^−/−^ C57BL/6 mice were produced by mating DNase II/IFN-I receptor (IR) double-knockout mice (strain B6.129-Dnase2a<tm1Osa> Ifnar1<tm1Agt>) (38–40) and C57BL/6 mice. DNase II/IFN-IR double-knockout mice, deposited by Shigekazu Nagata (Biochemistry & Immunology, Immunology Frontier Research Center, Osaka University) and Michel Aguent (ISREC-School of Life Sciences, EPFL), were provided by the RIKEN BioResource Center, Japan, through the National Bio-Resource Project of the MEXT, Japan.

### Animal experiments.

IFNAR^−/−^ C57BL/6 mice were bred and maintained in an environmentally controlled specific-pathogen-free animal facility of the NIID. Six- to 8-week-old female or male mice were used.

In infection experiments, each mouse was subcutaneously inoculated with 100 µl of virus solution (1.0 × 10^8^, 1.0 × 10^7^, 1.0 × 10^6^, and 1.0 × 10^5^ TCID_50_/ml each). For mock infection, the same volume of DMEM (placebo) was used. Each group consisted of 5 to 10 mice, which were given various doses of T-705, ribavirin, or placebo once a day by i.p. injection or per os (p.o.) using a stomach probe, just after subcutaneous inoculation of these mice with 1.0 × 10^6^ TCID_50_ of SFTSV (SPL010) in 100 µl DMEM. Treatments were commenced 1 h, 1 day, 2 days, 3 days, 4 days, or 5 days postinfection and continued for 5 days.

Blood samples (20 µl per animal) were obtained by tail vein puncture at intervals of 2 to 4 days over a period of 11 days (<4 blood drawings in total) for measurement of viral RNA levels. Body weight was recorded daily for 2 weeks, and each animal was monitored daily for the development of clinical signs, including hunched posture, ruffled fur, decreased activity, and response to stimuli, including neurological signs.

### Viral RNA titration.

The SFTSV genomic RNA was determined as previously described (32). Total RNA was prepared from 20 µl of blood samples using a High Pure viral RNA kit (Roche Life Science). Expression of the appropriate gene was estimated using a QuantiTect Probe RT-PCR kit (Qiagen, Hilden, Germany) according to the manufacturer’s protocol. Fluorescent signals were estimated using a LightCycler 96 (Roche Life Science). Statistics were performed using GraphPad Prism6 Software. One-way analysis of variance (ANOVA) with Bonferroni’s multiple-comparison test was used to compare viral RNA copies between ribavirin-, T-705- and placebo-treated groups.

### Histopathology and immunohistochemistry.

The mice treated with 300 mg/kg/day T-705 and placebo and with SFTSV were sacrificed using excess isoflurane at 12 and 4 days postinfection, respectively. The cervical lymph nodes, spleens, livers, and kidneys were collected for histopathological examination. The tissues were routinely processed and embedded in paraffin, sectioned, and stained with hematoxylin and eosin (H&E). Immunohistochemical (IHC) staining procedures were also performed to detect the SFTSV antigens in the paraffin-embedded sections as previously described (3). A rabbit polyclonal antibody against SFTSV NP (number 75) was used as primary antibody. Antigens were retrieved by hydrolytic autoclaving in citrate buffer (pH 6.0) for 10 min at 121°C. IHC staining was then performed using the EnVision/HRP immunodetection system (Dako, Glostrup, Denmark).
